# Genomic Analysis of Hepatitis B Virus Reveals Antigen State and Genotype as Sources of Evolutionary Rate Variation

**DOI:** 10.3390/v3020083

**Published:** 2011-01-25

**Authors:** Abby Harrison, Philippe Lemey, Matthew Hurles, Chris Moyes, Susanne Horn, Jan Pryor, Joji Malani, Mathias Supuri, Andrew Masta, Burentau Teriboriki, Tebuka Toatu, David Penny, Andrew Rambaut, Beth Shapiro

**Affiliations:** 1 Peter Medawar Building for Pathogen Research, Nuffield Department of Medicine, University of Oxford, South Parks Road, Oxford OX1 3SY, UK; 2 Fiji School of Medicine, Suva, Fiji; E-Mails: pryor.jan@gmail.com (J.P.); joji.malani@fnu.ac.fj (J.M.); 3 Department of Microbiology and Immunology, Rega Institute, K.U. Leuven 3000, Belgium; E-Mail: philippe.lemey@rega.kuleuven.be; 4 Wellcome Trust Sanger Institute, Wellcome Trust Genome Campus, Hinxton, CB10 1SA, UK; E-Mail: meh@sanger.ac.uk; 5 The Hepatitis Foundation of New Zealand, Ohope, Whakatane 3121, New Zealand; E-Mail: chris.moyes@bopdhb.govt.nz; 6 Max Planck Institute for Evolutionary Anthropology, Deutscher Platz 6, 04103 Leipzig, Germany; E-Mail: horn@eva.mpg.de; 7 School of Medicine and Health Sciences, University of Papua New Guinea, P.O. Box 5623, Boroko, Port Moresby, NCD, Papua New Guinea; E-Mails: sapuri@daltron.com.pg (M.S.); masta@daltron.com.pg (A.M.); 8 Nawerwere Hospital, Kiribati Ministry of Health, Tawara, Kiribati; E-Mails: dhsmhms@yahoo.com (B.T.); tebtoatu@yahoo.com (T.T.); 9 Allan Wilson Centre for Molecular Ecology and Evolution, Massey University, Palmerston North 4442, New Zealand; E-Mail: d.penny@massey.ac.nz; 10 Ashworth Laboratories, Institute of Evolutionary Biology, King’s Buildings, Edinburgh, EH8 3JT, UK; E-Mail: a.rambaut@ed.ac.uk; 11 Fogarty International Center, National Institutes of Health, Bethesda, MD 20892, USA; 12 Department of Biology, The Pennsylvania State University, University Park, PA 16802, USA

**Keywords:** hepatitis B virus, molecular clock, Bayesian phylogenetics

## Abstract

Hepatitis B virus (HBV) genomes are small, semi-double-stranded DNA circular genomes that contain alternating overlapping reading frames and replicate through an RNA intermediary phase. This complex biology has presented a challenge to estimating an evolutionary rate for HBV, leading to difficulties resolving the evolutionary and epidemiological history of the virus. Here, we re-examine rates of HBV evolution using a novel data set of 112 within-host, transmission history (pedigree) and among-host genomes isolated over 20 years from the indigenous peoples of the South Pacific, combined with 313 previously published HBV genomes. We employ Bayesian phylogenetic approaches to examine several potential causes and consequences of evolutionary rate variation in HBV. Our results reveal rate variation both between genotypes and across the genome, as well as strikingly slower rates when genomes are sampled in the Hepatitis B **e** antigen positive state, compared to the **e** antigen negative state. This Hepatitis B **e** antigen rate variation was found to be largely attributable to changes during the course of infection in the preCore and Core genes and their regulatory elements.

## 1. Introduction

Recent methodological advances in the genetic analysis of measurably evolving populations (MEPs [1]) have lead to the development of a wide range of models to investigate the underlying biological processes of viral evolution [2,3]. For example, it has become routine to use the temporal information, such as time of sampling, in genealogical analyses of viral data. These data provide a way to calibrate the rate of molecular evolution to calendar time, making it possible to test hypotheses about the timing and nature of specific evolutionary and epidemiological events. If the evolutionary rate is known, it is possible to estimate, for example, when a pathogen was first introduced into a particular species or population (e.g., [4]), to characterize variation in the rate of molecular evolution between viral subpopulations (e.g., [5]), or to reconstruct the demographic history of an epidemic (e.g., [6]).

For many viral data sets, the rate at which mutations accumulate is fast relative to the temporal period over which samples are isolated. The genetic diversity that accumulates over that time period can be used to inform estimates of the rate [1]. This is particularly true for RNA viruses, whose rapid rate of evolution makes them ideally suited for such analyses [7,8]. DNA viruses, alternatively, are thought to evolve more slowly, and consequently may be less suitable for evolutionary analyses spanning short time-frames (but see [9,10]).

Although Hepatitis B Virus (HBV) is classified as a DNA virus, it replicates through an RNA intermediary phase. HBV encodes its own reverse transcriptase, which, like those of rapidly evolving retroviruses, lacks proofreading capability, providing HBV the potential for high mutation rates. Nonetheless, previous research to quantify the tempo of HBV evolution have estimated rates at the lower range of RNA virus rates: around 1.4 × 10^−4^–5.7 × 10^−5^ substitutions per site per year [11–18]. While these rates are relatively slow, they are simultaneously too fast to reflect the suggested long-term association, and possibly co-speciation, between HBV strains and their primate hosts [19] and too slow to explain its extensive global genetic diversity [11–18].

The lack of resolution regarding HBV evolutionary rates is likely attributable to its complex biology. The HBV genome is highly constrained due to its small size (3200 base-pairs; bp), extensive overlapping reading frames and RNA secondary structure. These constraints result in high variability in substitution rates across the genome, for example, between the non-overlapping and overlapping coding regions. Nonetheless, the error-prone nature of its reverse transcriptase and frequent recombination at both the intra- and inter-genotype level [20] and between strains [21] can rapidly generate *de novo* diversity.

Strong host-pathogen interactions may also influence estimated rates of evolution in HBV, for example the regulation and expression of the Hepatitis B **e** antigen (HBeAg) and the Hepatitis B Core antigen (HBcAg). During early chronic infection, the HBeAg is expressed and stimulates regulatory T CD4 cells that suppress anti-viral T CD8 cell responses against HBcAg, which is a key antigen expressed on the hepatocyte cell walls [22,23]. HBeAg expression therefore assists in viral persistence, and prevents excessive immunological damage to the liver. In late chronic infection, the host frequently develops antibodies to HBeAg and/or the virus mutates, resulting in HBeAg negative (HBeAg−ve) infection. The mutations that can induce the HBeAg−ve status are collectively referred to as ‘preCore mutations’. Some preCore mutations eliminate HBeAg expression while others only modify expression. The most common of these mutations is a G to A substitution at nucleotide position (np) 1896 (G1896A), which creates a stop codon aborting the translation of the HBeAg and strengthens the secondary folding structure of the encapsulation signal (***ɛ***) on the viral RNA pre-genome, increasing viral replication [24–26]. The mutation also allows the host to mount an unregulated immune response against infected hepatocytes, typically leading to a lower viral load and less infectious state [18,27]. Indeed, observations that HBeAg−ve infections are frequently characterized by high nucleotide diversity compared to HBeAg+ve infections [12,16,18,28–30] may be explained by the increase in replication rate, combined with increase selection pressure in HBcAg. This situation would lead to variation in evolutionary rate during the course of infection, adding further complexity to rate estimation.

Here, we investigate these potential causes of evolutionary rate variation in HBV using a novel data set of 360 complete HBV genomes, representing several distinct genotypes sampled across the global distribution of the viruses and nearly 30 years of HBV evolutionary history (Table 1). We utilize the flexible phylogenetic analysis framework in BEAST [31] to design analyses that (1) allow pooling of molecular data (including recombinant lineages) without requiring that all model parameters be shared among every sequence; and (2) model variation in evolutionary rate both within the genome and between certain subsets of the data (such as HBeAg−ve sequences). We explore the patterns of evolutionary rate variation both within and between HBV genotypes and to test hypotheses about the influence of evolutionary constraints and changes in HBeAg status on rates of HBV evolution.

## 2. Results and Discussion

### 2.1. Variation in Evolutionary Rate between HBV Genotypes

Table 2 shows the estimated evolutionary rates for each of the nine genotype-specific data sets (data sets 1–3, 7, 9–13; Table 1). For each data set, we estimated evolutionary rates using both a strict and a relaxed (uncorrelated lognormal distribution, ucld) molecular clock. For the relaxed clock analyses, two mean rates are given: the ucld mean (*μ*), which is the mean of the rates on all the branches, and the weighted-mean (*μ_w_*), which is calculated by averaging the rates across all the branches in the genealogy, where each branch-specific rate is weighted according to the length (time) of each branch [32]. Relaxed clock results are not reported for the WH-C data set or for the WH-D HBeAg+ve data set as insufficient data were available to estimate a rate under this model. Further, no *μ_w_* results were given for the WH-Fa data sets, as the shared-rate approach (defined below) provides a different *μ_w_* for each family, as each family (subpopulation) informs its own, separate genealogy.

Although the 95% highest posterior density (HPD) intervals are often wide, the evolutionary rate varies considerably among the different genotypes analyzed. Most of our genotype-specific rate estimates fall within the range of rates estimated previously for HBV genomes (1.4 × 10^−4^–5.7 × 10^−5^); [11–18]. The among-host rate for HBV-A is the fastest rate estimated, with the 95% HPD intervals of both the strict and relaxed clock rates falling outside the previously published range. This is consistent with molecular assays that have shown a faster rate of replication for HBV-A compared to HBV-C and HBV-D [33]. In contrast to these results, Zehender *et al.* [34] reported rates estimated for the polymerase and surface antigen sequences of HBV genotypes A and D from a sample of patients in northwest Italy, in which they found a much faster rate for genotype D than for genotype A. The difference between this and our study may be due to the genomic region analyzed (our estimates are from complete genomes) or to population-specific differences in evolutionary rate.

The observed variation in evolutionary rate between different HBV genotypes may be explained by differences in their underlying biological properties. Evolutionary rate is a function of mutation rate and generation time (and thus replication rate), as well as the impact of natural selection. While each genotype has the same genomic structure and encodes the same polymerase enzyme, which probably results in similar mutational potential, they each have different primary routes of transmission, duration of infection, serological profiles and replication rates. For example, because of their geographic associations with developing nations and hyperendemicity, genotypes A-1, B, C and E are more likely to infect young children and infants, whereas genotypes A-2 D, F and H are more likely to be transmitted among adults [35–38]. Consequently the duration of infection and the time between transmission events for genotypes A-1, B, C and E are usually longer than for other genotypes. However, given the limited data currently available for many genotypes (for example, only 11 years of temporal data are currently available for genotype E) and the resulting large credible intervals, a larger sample size will be necessary to confirm this observation. Further, future insights into the quantification of the replication processes, selection pressures from the host immune system, and evolutionary dynamics of the genotypes as well as specific strains may better explain the observed rate variation between genotypes.

When genotype-specific data sets were analyzed assuming the lognormal relaxed clock model, *μ* was observed to be greater than *μ_w_* for each data set that contained a significant proportion of HBeAg−ve genomes. This result indicates a non-random distribution of rate variation along the trees. Such a pattern may emerge when a larger proportion of evolutionary changes occur along branches with short evolutionary time. Analysis of the maximum clade credibility (MCC) trees, on which it is possible to visualize the distribution of branch-rates summarized across all posterior trees, suggested that, when both HBeAg−ve and HBeAg+ve sequences were included in an alignment, the faster evolutionary rates were observed predominantly along branches leading to HBeAg−ve leaves (Figure 1). The highest rates occur almost exclusively along the short, terminal branches leading to HBeAg−ve sequences, suggesting that these lineages are evolving more rapidly than HBeAg+ve sequences.

### 2.2. Variation in Evolutionary Rate by HBeAg Status

To test whether HBeAg+ve genomes have slower average evolutionary rates than HBeAg−ve genomes, we performed additional analyses for the WH-D, AH-C, AH-D and AH-F data sets (data sets that contained both HBeAg+ve and −ve sequences) in which we excluded HBeAg−ve sequences (Table 3), thereby estimating a separate evolutionary rate only for HBeAg+ve sequences. Although confidence intervals sometimes overlapped, for each data set, the HBeAg+ve evolutionary rates were slower than those estimated from the full data sets. In addition, similar *μ* and *μ_w_* rates were obtained when only HBeAg+ve sequences were analyzed.

A change in status from HBVeAg+ve to HBVeAg−ve has been proposed previously to be associated with an increase in evolutionary rate [18]. To explore this further, we created four combined data sets according to HBeAg antigen status and whether the genomes were from WH (within host) or AH (among host) data sets (data sets 5, 6, 14 and 15; Table 1). We then estimated evolutionary rates under both a strict and relaxed clock model for each of these four data sets. For each analysis, we used the shared-rate approach so that each genotype informed its own separate genealogy while the other model parameters could be shared across the genotypes. For the relaxed clock analyses, all of the data in each data set were used to estimate the evolutionary rate. However, because each genotype has its own genealogy, the weighted mean evolutionary rate (*μ_w_*) can differ by genealogy, thus *μ_w_* is estimated separately for each subpopulation. For all four data sets, the standard deviations of the lognormal rate distributions in the relaxed clock analysis were skewed towards zero, indicating insufficient information to estimate rates under this model. However, in the strict clock analyses, although the HPD intervals overlap in the among-host data sets, the average rates estimated for both HBeAg+ve data sets were markedly slower than those of the corresponding HBeAg−ve data set (Table 4). This is most pronounced in the within-host data sets, which allow a direct comparison of the evolutionary rates of the same HBV infection before and after seroconversion from HBeAg+ve to HBeAg−ve. These results therefore add more weight to the hypothesis that evolutionary rate is affected by e-antigen status.

Rate variation according to HBeAg status may also have contributed to the variation observed between genotypes. Because of genotype-specific nucleotide variations, genotype A and specific strains of C and F seroconvert less frequently, or at a later stage of infection, to HBeAg−ve status (via the G1896A mutation) and are more likely to experience CURS and BCP mutations (the CURS-Core region comprises the Core Upstream Regulatory String (CURS) and Basal Core Promoter (BCP) region, which regulate translation of the HBeAg, as well as the HBeAg and HBcAg coding region) than are genotypes B, D, E, and H (a factor that is attributed to their increased virulence) [36,39–41]. While the G1896A mutation eliminates HBeAg expression and enhances replication, mutations in the CURS and BCP only modify HBeAg expression and do not enhance replication significantly [26,33]. And finally, genotype A has a stronger encapsulation signal structure which significantly increases replication [24,26,33]. These sequence variations, which result in different HBeAg serological profiles, will also induce different selection pressures from the host immune system.

### 2.3. Variation in Evolutionary Rate within HBV Genomes

To explore further the effect of e antigen status on evolutionary rate, we next partitioned the HBV genome into different regions, allowing each partitioned region to have its own evolutionary rate (the relative rate approach). Using the four data sets described above (data sets 4, 5, 14 and 15), we partitioned the genome in two ways. First, to test whether the structural composition of the genome influenced the evolutionary rate, we partitioned the genome into overlapping and non-overlapping regions. If overlapping regions are under stronger selective constraint than are non-overlapping regions, this analysis should result in a faster evolutionary rate for the non-overlapping partition regardless of the e-antigen status of the data set.

Second, we allowed different rates for the CURS-Core region (nucleotides 1645 to 2454), and the remainder of the HBV genome. This partitioning strategy thus allows us to estimate separately the evolutionary rate for the region influencing HBeAg expression and the remainder of the HBV genome.

The results of the partitioned analyses are presented in Tables 5 and 6. While we observe a trend suggesting that the overlapping region of the genome may evolve more slowly than the non-overlapping region, this trend is not significant (95% HPD intervals overlap for all four data sets). These results suggest that, while the existence of the overlapping reading frames remains an important consideration in HBV evolutionary models, standard analytical methods are sufficient to accommodate this rate variation and that excluding the overlapping regions is unnecessary.

A more interesting pattern emerges from the comparison of the CURS-Core region and the rest of the genome (Table 6). For both HBeAg+ve data sets, we observe no difference in evolutionary rate between the CURS-Core region and the rest of the genome. However, faster evolutionary rates are estimated for the CURS-Core region compared to the remainder of the genome for both HBeAg−ve data sets. This pattern is most pronounced in the WH-HBeAg−ve data set, where there is a log factor difference in evolutionary rate between the two partitions. The WH analysis is a direct comparison of sequences before and after seroconversion (see the within-host supplementary information), suggesting that the viral evolutionary rate is strongly influenced by the immunological status of the host.

Such localized rate variation is likely due to the immunological interactions of the HBeAg and the HBcAg (Hepatitis B virus core antigen). The HBeAg is a 29 amino acid (upstream) extension of HBcAg; both antigens express the same epitopes. In the early stages of chronic carriage, the HBeAg is expressed and stimulates regulatory T cells that suppress anti-viral T cell responses against the HBcAg, a key antigen expressed on the hepatocyte cell walls essential for virion formation [22,23]. HBeAg expression therefore assists in viral persistence, prevents excessive immunological damage to the liver, and minimizes host immune selection pressure on the HBcAg. However, in late chronic infection, hosts frequently develop antibodies to HBeAg (anti-HBeAg) whereupon the virus mutates, resulting in a HBeAg negative (HBeAg−ve) infection. When this occurs, translation and expression of the HBcAg, as well as the polymerase, is enhanced, leading to increased viral replication [26,33], and the regulation of the immune response is lifted. The combined result is a higher rate of replication and stronger immune selection pressure, which in turn will result in greater sequence variation. Consequently, under these conditions, the CURS-Core region is under strong selection pressure from the host immune system and mutations in the HBcAg can provide a selective advantage to the virus, enabling viral persistence.

### 2.3. Modeling the Influence of HBeAg Serological State on Evolutionary Rate

Finally, we performed an additional series of analyses in which we directly assessed the influence of **e** antigen status on the evolutionary rate. We used several novel modifications of the *delta model* [42], which models additional substitutions along specific branches in a phylogeny. Specifically, we compare a *specific delta*, in which branches leading to HBeAg−ve leaves are allowed to evolve more rapidly than the other branches in the tree, a *general delta*, in which all terminal branches are allowed to evolve more quickly than internal branches (this accommodates extra substitutions that may be present in each branch, for example as may occur while weakly deleterious mutations are in the process of being removed from the population), and a *no delta*, in which all branches evolve at the same rate. Delta analyses were performed on the three genotype-specific data sets for which sufficient numbers of both HBeAg+ve and HBeAg−ve sequences were available (WH-D, AH-C and AH-D; data sets 2, 9 and 10).

Results of the delta model analyses are presented in Table 7. For the AH-D and WH-D data sets, Bayes factors indicate strong support favoring the specific delta model over the general delta model (AH-D 2lnB01 = 33.56; WH-D 2lnB01 = 68) and the specific delta model over the no delta model (AH-D 2lnB01 = 22.30; WH-D 2lnB01 = 66). There is also marginal support (AH-D 2lnB01 = 11.2; WH-D 2lnB01 = 2.44) for the no delta model over the general delta model. These results suggest an increased rate of substitution only along terminal branches leading to HBeAg−ve sequences. When these excess substitutions are accommodated by the specific delta model the global evolutionary rate slows to a value comparable to that estimated for the AH-D data set without HBeAg−ve sequences. The specific delta parameter therefore appears to have absorbed the rate difference observed between HBeAg−ve and HBeAg+ve sequences enabling a more accurate estimation of the long-term evolutionary rate.

For the AH-C dataset, we find only moderate support favoring the specific delta over the general delta (2lnB01 = 4.6) and no support for favoring the specific delta over the model without delta (2lnB01 = −0.67). This difference between the genotype D and genotype C may be due to their different susceptibility to CURS BCP and preCore mutations. We defined HBeAg−ve status by serological test results and the presence of the G1896A mutation. However, mutations in the CURS and BCP region can reduce HB**e** antigen expression, resulting in a similar immunological and virological state to HBeAg−ve serological status. The genomic sequence and structure of genotype C is less susceptible to the G1896A mutation and more susceptible to CURS and BCP mutations than is genotype D [43], which may explain the results observed here.

## 3. Conclusions

In this work, we use several different Bayesian inference models to estimate the evolutionary rate from a broad geographic sample of HBV complete genomes. We compare differences in evolutionary rate between genotypes, between regions of the genome, and between viruses with different serological states. We found that regardless of genotype, a change in serological state from HBeAg+ve to HBeAg−ve coincides with an increase in evolutionary rate. In addition, we found that neither genotype nor genomic region significantly influences the estimated rate in our sample of HBV. We also note that in comparing WH and AH data sets, inference was often more straightforward for data sets where the viral strains were most closely related (WH). Given that AH data sets will have significantly more variables that the models will need to accommodate, this result is perhaps unsurprising. Nonetheless, this result highlights the significant evolutionary variation that is known to exist both within and between viral data sets, clearly demonstrating how this variation can confound phylogenetic and phylogeographic analyses.

Differences between HBeAg positive and negative serological states have been recognized at both the clinical and nucleotide sequence level for some time. For example, it is known that the clinical prognosis for HBeAg−ve individuals with low viral titer is far better than for HBeAg+ve individuals with high viral titer [41] and it is has been reported that sequence divergence, as a whole, is greater in HBeAg−ve sequences [18]. Our results illustrate that including HBeAg−ve sequences in phylogenetic analysis of individual genotypes is likely to bias evolutionary rate estimates, and that these biases can be inconsistent between genotypes. We therefore recommend that future analyses of the global distribution of HBV genotypes are careful to appropriately model HBeAg status.

## 4. Methods

### 4.1. Data Collection

Pacific Island HBV positive samples were either provided by persons as listed in the acknowledgements or were identified from a tri-nation screening program involving Papua New Guinea, Fiji and Kiribati to investigate HBV vaccine escape, viral diversity, and phylogeography in South Pacific island countries. Ethical permission was obtained from each country through the appropriate committees. Viral DNA was extracted from serum samples using the High Pure Viral Nucleic Acid™ Kit—for the isolation of nucleic acids for PCR or RT-PCR, as per manufacturers’ instructions. Complete HBV genomes were PCR-amplified in two overlapping fragments as described by Gunther *et al*. [30] using primers HB1839R (GCTTGAGCTCTTCAAAAAGTTGCATGGTGCTGG)-HB1877F (GCTTGAGCTCTTCTTTTTCACCTCTGCCTAATCA) for the complete genome, and HB1611 (CGCTTCACCTCTGCACGTCGCA)-HB2313 (YTCCGGAAGTGTTGATARGATAGG) for the smaller overlap. Roche Expand High Fidelity PCR-plus™ enzyme was used as per the kit recommendations. The genomic PCR DNA fragments were either sequenced directly or used as template in a second-round nested PCR to generate shorter fragments (0.8–1.6 kb). The times and temperatures for the extension and primer annealing steps varied slightly depending on the expected length of the fragment and the desired annealing temperature of the primers, respectively. In total, 112 complete HBV genomes with known sampling date were sequenced using this approach (GenBank accession numbers HQ700439-HQ700440, HQ700442-HQ700443, HQ700445-HQ700448, HQ700452, HQ700454-HQ700456, HQ700458-HQ700459, HQ700461-HQ700462, HQ700464, HQ700466-HQ700470, HQ700472-HQ700474, HQ700477-HQ700478, HQ700480-HQ700481, HQ700484-HQ700486, HQ700488-HQ700490, HQ700492-HQ700527, HQ700530-HQ700541).

To construct expanded global data sets, we obtained an additional 228 sequences representing all information-complete HBV genomes available in Genbank as of April 2007. Sequences were regarded as information-complete when data for collection dates, serological status (HBeAg and anti-HBeAg), and epidemiological relationships between samples could be compiled, either via direct communication with the authors or from the relevant publications. An additional 85 sequences were obtained in July 2010 to increase the number of sequences for the HBV genotypes A, E, F, and H to greater than, or equal to, 20 each. (A detailed description of each genome sequence is provided as Supplementary Material Table 1).

The complete data set of 425 HBV genomes was subdivided into two major categories. The first group of data sets includes longitudinal samples collected from within individual hosts and short-term transmission (pedigree) data (WH data sets). We compiled four WH data sets: recombinant genotype BC (WH-BC; [16]), genotype C (WH-C; this study), genotype D (WH-D; [15,16], this study), and a combined pedigree dataset from three epidemiologically unlinked families (WH-Fa; [44]). To investigate the effect of HBeAg status on the evolutionary rate of HBV and to investigate rate variation between different regions of the genome, sequences from all four WH data sets were pooled and used to construct separate WH-HBeAg−ve and WH-HBeAg+ve data sets. Second, to address among-host evolution, we compiled data sets comprising epidemiologically unrelated genomes (AH data sets). Based on the number of genomes available, we were able to compile six genotype-specific AH data sets: genotypes A, C, D, E, F and H. As above, the AH sequences were then pooled and used to compile AH-HBeAg+ve and AH-HBeAg−ve data sets for further analysis (Table 1).

### 4.2. Detecting Recombinants

To detect inter-genotype recombinant HBV genomes, we used a modified version of the Oxford Hepatitis B Virus Genotyping Tool that included representative simian HBV strains. This tool is available on the BioAfrica web site [45]. Within-genotype recombination was assessed initially using the Phi-test, which uses the pairwise homoplasy index to assess whether the substitution patterns deviate significantly from clonality [46,47]. If this test revealed significant evidence for recombination, the program 3SEQ was used to identify putative recombinants [47]. In the case where these two intra-genotype methods were inconsistent, we also investigated reticulate evolution using SplitsTree, [48]. The recombinants identified using this approach are listed in the supplementary material (Supplementary Table 2). All putative recombinants except the pedigree genotype rBC sequences (WH-BC) were excluded from further analysis.

### 4.3. Inferring Evolutionary Rates

Evolutionary rates were estimated using Bayesian Monte Carlo Markov Chain (MCMC) analyses as implemented in BEAST [31] using the Hasegawa-Kishino-Yano model of evolution (HKY85) with a proportion of invariant sites, and a constant population size demographic model [49]. For the WH-Fa data set, the time to the most recent common ancestor (MRCA) for each child lineage and any other lineage was constrained to be earlier than the date of birth of the child. In addition, the root of the complete familial genealogies was constrained to be younger than the date of birth of the mother. In essence, this represents a full probabilistic genealogical estimation procedure for a previously reported pedigree rate estimation problem [50].

For all data sets, both strict and relaxed (uncorrelated lognormal distribution; [32]) clocks were applied in separate analyses. MCMC chains were run until stationarity was achieved, as evaluated using Tracer [51]. Rate variation between specific genomic regions was modeled using relative rate parameters. Novel models developed for this analysis are presented below. Trees were summarized using TreeAnnotator and visualized in FigTree [52].

### 4.4. BEAST Analyses

BEAST [31] is a flexible, coalescent-based platform for phylogenetic and genealogic inference. In addition to more standard coalescent models described above, we take advantage of this flexibility to test the hypotheses evaluated above using three additional models.

#### 4.4.1. Shared-rate Approach

The WH-BC and WH-Fa datasets include inter-genotype recombinant B-C sequences. Since the shared ancestry of non-recombinant and recombinant lineages cannot be modeled with a strictly bifurcating tree, we allow each within-host data set and each family to act as a ‘subpopulation’ and have its own genealogy, while sharing the rate across the genealogies. This shared-rate approach was used for all analyses of the WH-BC and WH-Fa data sets, as well as for the analyses of the larger HBeAg+ve and HBeAg−ve data sets, where separate genealogies were estimated for each of the genotypes within the larger data set.

This approach shares the substitution rate, the transition/transversion ratio and the proportion of invariant sites across the individual genealogies, although the genealogies are allowed to be different for each subset of sequences. Under the relaxed clock model, for each separate genealogy branch-specific rates are sampled from underlying lognormal distributions with the same mean but different standard deviations. This approach is conceptually similar to the likelihood-based approaches developed by Rodrigo *et al.* [5,53]; the Bayesian model implementation is, however, similar to the ‘unlinked model’ of Lemey *et al.* [53].

#### 4.4.2. Relative Rates Approach

To investigate rate variability across the genome, we incorporated relative rates across different alignment partitions. This approach uses a relative rate factor, *r_j_*, for *m* different genome regions, In combination with the shared-rate approach, *r_j_* is the same for the same genome region in all the *n* subpopulations. Using this model, we evaluated the relative rate differences between overlapping and non-overlapping genome regions, as well as between the CURS, BCP preCore and the Core regions (1645–2454 nt) and the rest of the HBV genome.

#### 4.4.3. Specific Delta Model

We hypothesize that the change in antigen state from HBeAg+ve to HBeAg−ve can result in a change in the evolutionary rate. To test this, we implement a model that allows a subset of lineages to evolve at a different rate compared to the rest of the tree.

Ho *et al.* [42] provide a Bayesian model that allowed an extra amount of substitutions to occur along terminal branches. This model, referred to as the *delta model*, was used to estimate and accommodate DNA damage in ancient DNA sequences. We extend the delta model to allow extra substitutions to occur along *specified* terminal branches in the tree, applied here to HBeAg−ve sequences. The fit of this model (‘specific delta’) was compared to the fit of a model that allowed the same additional amount of substitutions for all tips (‘general delta’) and a model that did not allow for additional substitutions at the tips (‘no delta’). Models were compared using Bayes factors (specifically, two times the log of the Bayes factor, 2lnB01, where B01 = P(Model 0|Data)/ P(Model 1|Data)) [54]. Comparisons were performed on the three data sets that had sufficient HBeAg−ve and HBeAg+ve sequences (WH-D, AH-D and AH-C datasets).

## Supplementary Materials



## Figures and Tables

**Figure 1.**
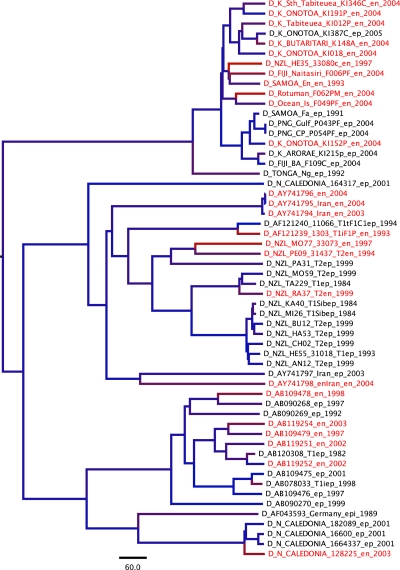
Maximum clade credibility (MCC) resulting from the analysis of the Genotype D between-host data set. Colors along the branches indicate relative rates, from a scale of blue (the most slowly-evolving branches) to red (the most rapidly-evolving branches). Taxon labels of the most rapidly evolving branches are highlighted in red.

**Table 1.** Details of the 15 data sets used in the analyses described in the main text. When more than one “subpopulation” is included in a data set, each informs its own genealogy using the shared-rate approach.

	**Data sets**		**Data set name**	**Genotypes**	**Number of sequences**	**HBeAg+ve**	**HBeAg−ve**	**Recombinants strains**	**Subpopulations per data set**
Serially sampled Within Host and Family Transmission sequences	1	Within Host Genotype C	WH-C	C	11	9	2	0	4
	2	Within Host Genotype D	WH-D	D	27	21	6	0	13
	3	Within Host recombinant sequences of Genotypes B and C	WH-BC	rBC	16	1	15	-	8
	4	Family Transmission sequences of Genotype D and recombinant sequences of Genotypes B and C	WH-Fa	D, rBC	13	7	6	0	3
	5	HBeAg+ve Within Host and Family Transmission sequences of Genotypes C, D and recombinant sequences of B and C	WH-HBeAg+ve	C, D, rBC	54	n/a	n/a	0	3
	6	HBeAg−ve Within Host and Family Transmission sequences comprised of recombinant genotype B and C	WH-HBeAg−ve	rBC	34	n/a	n/a	0	9

Among Host epidemiologically unrelated sequences	7	Among Host Genotype A	AH-A	A	37	37	0	5	n/a
	8	Among Host Genotype B	AH-B	B	15	5	10	-	n/a
	9	Among Host Genotype C	AH-C	C	63	18	18	-	n/a
	10	Among Host Genotype D	AH-D	D	56	25	25	-	n/a
	11	Among Host Genotype E	AH-E	E	49	45	45	0	n/a
	12	Among Host Genotype F	AH-F	F	35	26	26	4	n/a
	13	Among Host Genotype H	AH-H	H	22	22	22	0	n/a
	14	Among Host HBeAg+ve	AH-HBeAg+ve	C, D	76	n/a	n/a	n/a	n/a
	15	Among Host HBeAg−ve	AH-HBeAg−ve	C, D	43	n/a	n/a	n/a	n/a

**Table 2.** Evolutionary rates estimated assuming the strict molecular clock and the uncorrelated lognormal (UCLD) relaxed clock. The difference between the mean rate and the weighted mean rate is explained in the main text. The WH-C HBeAg+ve data set contained insufficient information to estimate an evolutionary rate under the relaxed clock, and these results are not reported here.

**Data Set**	**Antigen state**	**Strict Clock**		**UCLD Relaxed Clock**		**UCLD Relaxed Clock**	
		**Mean**	**95% HPD**	**UCLD Mean**	**95% HPD**	**Weighted Mean**	**95% HPD**
AH-A	HBeAg+ve	6.01E-04	4.07E-04–7.83E-04	8.60E-04	4.34E-04–1.43E-03	8.04E-04	4.41E-04–1.26E-03
AH-C	HBeAg+ve and −ve	1.23E-04	2.81E-05–2.12E-04	2.00E-04	5.41E-05–3.61E-04	1.88E-04	5.09E-05–3.34E-04
AH-D	HBeAg+ve and −ve	1.01E-04	4.57E-05–1.53E-04	1.21E-04	1.83E-05–2.27E-04	9.39E-05	1.87E-05–1.77E-04
AH-E	HBeAg+ve	1.94E-04	7.98E-06–3.75E-04	9.29E-04	1.81E-05–2.018E-03	6.97E-04	1.41E-04–1.28E-03
AH-F	HBeAg+ve and −ve	5.29E-04	3.49E-04–6.85E-04	1.11E-03	5.18E-04–1.76E-03	8.39E-04	4.44E-04–1.20E-03
AH-H	HBeAg+ve	4.39E-05	3.97E-08–1.11E-04	2.88E-04	6.48E-07–6.67E-04	1.75E-04	3.77E-06–3.54E-04
WH-BC	HBeAg−ve	9.55E-05	4.80E-05–1.52E-04	1.12E-04	1.40E-07–2.21E-04	9.63E-05	9.31E-06–1.80E-04
WH-C	HBeAg+ve and −ve	1.15E-04	3.09E-05–2.13E-04		-		-
WH-D	HBeAg+ve and −ve	1.36E-04	9.40E-05–1.80E-04	1.17E-04	3.49E-05–2.08E-04	5.78E-05	1.08E-05–1.16E-04

**Table 3.** Evolutionary rates estimated from four data sets restricted only to HBeAg+ve sequences.

**Data Set**	**Antigen state**	**Strict Clock**		**UCLD Relaxed Clock**		**UCLD Relaxed Clock**	
		**Mean**	**95% HPD**	**UCLD Mean**	**95% HPD**	**Weighted Mean**	**95% HPD**
AH-C	HBeAg+ve	8.76E-05	1.48E-06–1.79E-04	2.47E-04	1.20E-05–4.84E-04	2.29E-04	1.15E-05–4.37E-04
AH-D	HBeAg+ve	5.93E-05	1.26E-04–1.01E-04	7.60E-05	1.90E-05–1.43E-04	6.73E-05	1.37E-05–1.23E-04
AH-F	HBeAg+ve	1.80E-04	4.78E-05–3.41E-04	5.61E-04	2.06E-05–1.15E-03	4.10E-04	3.22E-05–7.62E-04
WH-D	HBeAg+ve	3.74E-05	8.83E-06–7.11E-05	-		-	

**Table 4.** Evolutionary rates estimated for the combined within-host and among-host data sets assuming a strict molecular clock and the shared-rate approach.

	**HBeAg−ve**		**HBeAg+ve**	
	**Mean**	**95% HPD**	**Mean**	**95% HPD**
Within-host	1.10E-04	8.23E-05–1.41E-04	2.60E-05	1.49E-05–3.75E-05
Among-host	2.01E-04	4.88E-05–3.32E-04	6.10E-05	1.97E-05–1.02E-04

**Table 5.** Evolutionary rates for complete genomes, overlapping regions and non overlapping regions estimated from the combined genotype data sets.

		**Strict Clock**		**UCLD Relaxed Clock**	
		**Mean**	**95% HPD**	**UCLD Mean**	**95% HPD**
Within-host HBeAg +ve	complete genome	2.60E-05	1.49E-05–3.75E-05	4.43E-05	2.24E-05–6.96E-05
	nonoverlapping	3.30E-05	1.89E-05–4.83E-05	5.87E-05	2.94E-05–9.31E-05
	overlapping	1.71E-05	9.38E-06–2.47E-05	3.07E-05	1.36E-05–4.4E-05

Within-host HBeAg −ve	complete genome	1.10E-04	8.23E-05–1.41E-04	1.17E-04	8.40E-05–1.53E-04
	nonoverlapping	1.34E-04	9.59E-05–1.72E-04	1.42E-04	9.94E-05–1.88E-04
	overlapping	8.66E-05	6.06E-05–1.41E-04	9.30E-05	6.41E-05–1.16E-04

Among-host HBeAg +ve	complete genome	6.10E-05	1.97E-05–1.02E-04	6.20E-05	2.09E-05–1.06E-04
	nonoverlapping	8.36E-05	3.64E-05–1.38E-04	8.26E-05	2.81E-05–1.41E-04
	overlapping	4.29E-05	1.56E-05–6.77E-05	4.25E-05	1.52E-05–7.37E-05

Among-host HBeAg −ve	complete genome	2.01E-04	4.88E-05–3.32E-04	1.89E-04	3.96E-05–3.44E-04
	nonoverlapping	2.52E-04	8.74E-05–4.37E-04	2.34E-04	4.47E-05–4.24E-04
	overlapping	1.56E-04	5.36E-05–2.71E-04	1.45E-04	3.01E-05–2.65E-04

**Table 6.** Evolutionary rates for complete genome, non CURS-Core region and CURS-Core region estimated from the combined genotype data sets.

		**Strict Clock**		**UCLD Relaxed Clock**	
		**Mean**	**95% HPD**	**UCLD Mean**	**95% HPD**
Within-host HBeAg +ve	complete genome	2.58E-05	1.53E-05–3.74E-05	4.77E-05	2.55E-05–7.94E-05
	non CURS-Core	2.62E-05	1.52E-05–3.76E-05	4.81E-05	2.47E-05–7.95E-05
	CURS-Core	2.49E-05	1.26E-05–3.68E-05	4.67E-05	2.06E-05–7.77E-05

Within-host HBeAg −ve	complete genome	1.09E-04	8.13E-05–1.39E-04	1.24E-04	8.71E-05–1.62E-04
	non CURS-Core	8.61E-05	6.35E-05–1.09E-04	9.72E-05	6.85E-05–1.31E-04
	CURS-Core	1.79E-04	1.21E-04–2.35E-04	2.02E-04	1.37E-04–2.72E-04

Among-host HBeAg +ve	complete genome	5.99E-05	2.32E-05–9.92E-05	6.31E-05	2.20E-05–1.00E-04
	non CURS-Core	6.24E-05	2.27E-05–1.02E-04	6.58E-05	2.30E-05–1.05E-04
	CURS-Core	5.23E-05	1.87E-05–8.66E-05	5.50E-05	2.01E-05–8.96E-05

Among-host HBeAg −ve	complete genome	1.95E-04	1.89E-05–3.36E-04	2.00E-04	6.32E-05–3.42E-04
	non CURS-Core	1.78E-04	2.34E-05–3.13E-04	1.83E-04	5.38E-05–3.08E-04
	CURS-Core	2.44E-04	2.60E-05–4.29E-04	2.51E-04	6.91E-05–4.25E-04

**Table 7.** Evolutionary rate estimates under the no delta, specific delta and general delta models rate for the WH-D, AH-D and AH-C data sets.

**Datasets**	**Model**	**Clock Rate**	**95% HPD**	**Delta Distribution**	**95% HPD**	**Log P**
	No delta	1.36E-04	9.40E-05–1.80E-04	-	-	−6319.237
	General Delta	1.31E-04	8.52E-05–1.74E-04	skewed to zero	-	−6320.459
WH- D	Specific Delta	4.35E-05	1.26E-05–7.41E-05	5.62E-03	4.09E-03–6.94E-03	−6286.171
	No delta	1.02E-04	4.57E-05–1.53E-04	-	-	−10732.539
	General Delta	9.58E-05	4.30E-05–1.50E-04	5.16E-04	1.65E-04–9.26E-04	−10738.168
AH- D	Specific Delta	6.74E-05	1.56E-05–1.14E-04	2.54E-03	1.77E-03- 3.24E-03	−10721.39
	No delta	1.20E-04	2.98E-05–1.95E-04		-	−19005.325
	General Delta	8.48E-05	2.07E-05–1.54E-04	1.66E-03	8.07E-04–2.44E-03	−19007.966
AH- C	Specific Delta	8.45E-05	2.29E-05–1.56E-04	1.90E-03	7.40E-04–3.11E-03	−19005.66
